# Simulation-Based Optimization of Sampling Schedules for Model-Informed Precision Dosing of Once-Daily and 4-Times-Daily Busulfan in Pediatric Patients

**DOI:** 10.1097/FTD.0000000000001217

**Published:** 2024-06-14

**Authors:** Khalil Ben Hassine, Youssef Daali, Yvonne Gloor, Tiago Nava, Yves Théorêt, Maja Krajinovic, Henrique Bittencourt, Chakradhara Rao Satyanarayana Uppugunduri, Marc Ansari

**Affiliations:** * CANSEARCH Research Platform for Pediatric Oncology and Hematology, Department of Pediatrics, Gynecology, and Obstetrics, Faculty of Medicine, University of Geneva, Geneva, Switzerland;; † Division of Clinical Pharmacology and Toxicology, University Hospital of Geneva, Geneva, Switzerland;; ‡ Faculty of Medicine & Sciences, University of Geneva, Geneva, Switzerland;; § Charles-Bruneau Cancer Center, CHU Sainte-Justine Research Center, Montreal, Quebec, Canada;; ¶ Department of Pediatrics, Faculty of Medicine, University of Montreal, Montreal, Quebec, Canada;; ║ Clinical Pharmacology Unit, CHU Sainte-Justine, Montreal, Quebec, Canada; and; ** Division of Pediatric Oncology and Hematology, Department of Women, Child, and Adolescent, University Hospital of Geneva, Geneva, Switzerland.

**Keywords:** busulfan, therapeutic drug monitoring, model-informed precision dosing, limited sampling strategy, pediatric, hematopoietic stem cell transplantation

## Abstract

**Background:**

Therapeutic drug monitoring (TDM) is crucial in optimizing the outcomes of hematopoietic stem cell transplantation by guiding busulfan (Bu) dosing. Limited sampling strategies show promise for efficiently adjusting drug doses. However, comprehensive assessments and optimization of sampling schedules for Bu TDM in pediatric patients are limited. We aimed to establish optimal sampling designs for model-informed precision dosing (MIPD) of once-daily (q24h) and 4-times-daily (q6h) Bu administration in pediatric patients.

**Methods:**

Simulated data sets were used to evaluate the population pharmacokinetic model-based Bayesian estimation of the area under the concentration–time curve (AUC) for different limited sampling strategy designs. The evaluation was based on the mean prediction error for accuracy and root mean square error for precision. These findings were validated using patient-observed data. In addition, the MIPD protocol was implemented in the Tucuxi software, and its performance was assessed.

**Results:**

Our Bayesian estimation approach allowed for flexible sampling times while maintaining mean prediction error within ±5% and root mean square error below 10%. Accurate and precise AUC_0–24h_ and cumulative AUC estimations were obtained using 2-sample and single-sample schedules for q6h and q24h dosing, respectively. TDM on 2 separate days was necessary to accurately estimate cumulative exposure, especially in patients receiving q6h Bu. Validation with observed patient data confirmed the precision of the proposed limited sampling scenarios. Implementing the MIPD protocol in Tucuxi software yielded reliable AUC estimations.

**Conclusions:**

Our study successfully established precise limited sampling protocols for MIPD of Bu in pediatric patients. Our findings underscore the importance of TDM on at least 2 occasions to accurately achieve desired Bu exposures. The developed MIPD protocol and its implementation in Tucuxi software provide a valuable tool for routine TDM in pediatric hematopoietic stem cell transplantation.

## Abstract 2

Supplemental Digital Content is Available in the Text.

## INTRODUCTION

Therapeutic drug monitoring (TDM) for busulfan (Bu) represents a significant advancement in optimizing hematopoietic stem cell transplantation outcomes. The relevance of TDM-guided targeted dosing of Bu has been demonstrated, showing benefits in both target achievement and clinical outcomes.^1–9^ Bu is an alkylating agent commonly administered either once daily (every 24 hours, q24h) or 4 times daily (every 6 hours, q6h) through iterative intravenous infusions over 4 days in typical myeloablative regimens. This agent stands as an exemplary candidate for routine TDM application due to several contributing factors.^10,11^ Bu is noted for its significant exposure-outcome and toxicity associations, particularly in pediatric populations.^6,12,13^ Compelling data in this population reveal an optimal exposure range associated with the highest probability of event-free survival, while maintaining an acceptable toxicity level, corresponding to a cumulative AUC of 78–101 mg·h/L.^6^

Moreover, Bu exhibits substantial interindividual pharmacokinetic (PK) variability, as to its narrow therapeutic window, as well as intraindividual exposure variability (IOV). IOV of Bu mostly manifests as a decrease in clearance (CL) throughout treatment,^14–19^ although a minority of patients may display unchanged or increased CL.^15,18^ The evidence of unpredictable IOV in Bu PK supports the prescription of TDM on multiple occasions and necessitates the use of models capable of predicting these CL changes. Consequently, several studies have incorporated a decrease in CL throughout treatment into Population PK (PopPK) models.^14,16,17,19–21^

Limited sampling strategies (LSSs) have garnered significant attention in TDM of Bu and other drugs due to their potential for practical, efficient, and precise dose adjustment with minimal inconvenience.^22^ LSS involves estimating PK parameters using fewer blood samples, thereby reducing the burden on patients, healthcare providers, and laboratory staff.^23^ Given the IOV in Bu CL, it remains uncertain whether monitoring a single dose (eg, solely on the first day of treatment, as commonly practiced) is adequate for achieving the desired cumulative exposure to Bu, particularly in children who exhibit even greater variability than adults.^24^ As Bu conditioning is typically brief, sampling schemes must be meticulously optimized to ensure accurate attainment of a narrow-targeted therapeutic window, thus enhancing the likelihood of treatment success.

We hypothesized that this success could be attained through the combined implementation of precise initial dosing of Bu along with an optimized and standardized TDM protocol. We previously focused on personalizing the initial dosing of Bu in pediatric patients using a PopPK model, which incorporates individual covariates, such as genetic variants of the *GSTA1* promoter.^14^ The aim of this study was to determine the optimal sampling designs for model-informed precision dosing (MIPD) of once-daily and 4-times-daily Bu administration before pediatric hematopoietic stem cell transplantation. The optimized designs consider the number and timing of samplings for each dose and the frequency of TDM over multiple days.

## METHODS

**FIGURE 1.**
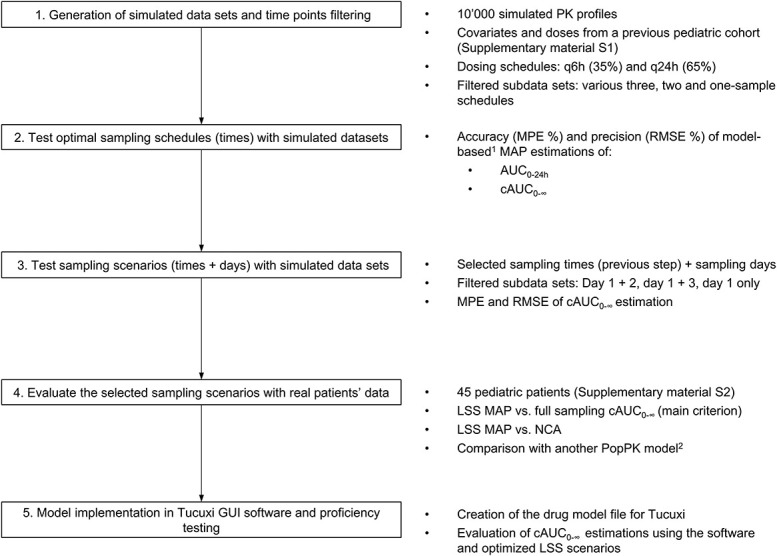
Overall methodology workflow for the study. AUC_0–24h_, area under the plasma concentration curve, up to 24 hours after treatment start: cAUC_0–∞_, cumulative AUC across the treatment; GUI: graphical user interface; MPE %: relative MPE; q6h: four-times-daily dosing (every 6 hours); q24h: once-daily dosing (every 24 hours); RMSE %: relative root mean square error. ^1^Using our previously published model.^14^ ^2^Using a previously published and clinically evaluate model by Shukla et al.^21^

The workflow depicted in Figure 1 outlines the analysis process. For clarity, the term “sampling schedules” refers to the specific times at which samples are collected for PK monitoring of each dose; “sampling scenarios” denote the combinations of sampling times and days on which TDM is conducted.

### Generation of the Simulated Data Set

The simulated data set comprised administered doses, infusion durations, and patient covariates obtained from individuals in the validation data set of our previously published model (n = 100).^14^ **Supplemental Digital Content 1** (see **Supplementary Material S1**, http://links.lww.com/TDM/A747) provides an overview of the demographic characteristics and administered doses for these patients. Stochastic simulations were employed to generate 100 replicates of PK profiles for each individual by sampling from the distribution of previously published PK model parameters.^14^ Phoenix NLME (Version 8.2, Certara, Princeton, NJ) facilitated these simulations. To generate realistic simulated data, we opted to simulate “Cobs” concentration values in Phoenix software, accounting for random residual variability. This approach yielded concentration profiles with inherent noise representative of analytical imprecision. We created subdatasets corresponding to the sampling schedules and scenarios under investigation by extracting relevant time points from the original simulated data set. R (version 4.1.1, CRAN) with the *tidyverse* package was utilized for the preparation of simulated data sets.

### Selection of Limited Sampling times

The accuracy and precision of model-based Bayesian maximum a posteriori (MAP) estimation of exposure were assessed using the simulated data sets. These comprised routinely feasible limited sampling time combinations (schedules) over the first 3 consecutive days of treatment (1 occasion per day for q6h dosing). MAP estimations were conducted using the previously published PopPK model.^14^ AUC_0–24h_ and cumulative AUC (cAUC_0–∞_) with LSS schedules were obtained by integrating the MAP concentration profiles in Phoenix NLME. In addition, a *priori* estimation from the covariate model without concentration input was also evaluated. The evaluation criteria for the sampling schedules were the relative mean prediction error (MPE) for accuracy and the relative root mean square error (RMSE) for precision, which were calculated according to Equations 1 and 2:

(1) $$\text{MPE}\;\%=\frac{1}{n}\sum \frac{\text{Pred}-\text{Ref}}{\text{Ref}}\times 100$$

(2) $$\text{RMSE}\;\%=\sqrt{\frac{1}{n}\sum {\left(\frac{\text{Pred}-\text{Ref}}{\text{Ref}}\right)}^{2}}\times 100$$

Pred are the PK exposure values predicted by MAP using the limited sampling schedule/scenario, and Ref are the reference AUC values of the simulated full concentration–time profiles.

To ensure reliability, we defined stringent criteria for selecting sampling schedules. Schedules with MPE outside ±5% interval and RMSE >10% were deemed noncompliant. We tested the optimal design software PFIM interface (version 4.0) to determine whether Fisher information matrix-based calculations could be a reliable alternative to time-consuming simulations for an optimal LSS design.^25^ Bayesian design optimization was performed using the Federov–Wynn algorithm. As the PFIM interface 4.0 cannot handle covariates, typical PK parameter values were used in calculations.

### Selection of Sampling Day (Scenarios)

Using the sampling schedules selected in the previous step, we assessed various sampling scenarios, including sampling on day 1, day 1 + 2, or day 1 + 3. For q6h dosing, the sampling of 1 dosing occasion per day was considered during the evaluation of the scenarios. In addition, a scenario with full sampling (ie, 7 and 8 samples for q6h and q24h, respectively) on the first day only was tested. We compared the MPE and RMSE of the cAUC_0–∞_ to evaluate the performance of the different scenarios.

### Evaluation of the Strategies on the Real Patients' Data

Once optimal sampling scenarios were determined using the simulated data sets, we assessed the concordance between cAUC_0–∞_ obtained with limited sampling versus full sampling (the reference). This assessment utilized clinically observed plasma concentrations from a subset of patients with extensive sampling data available (Total n = 45, q6h n = 23, q24h n = 22). Bland–Altman analyses were employed for comparisons. These patients participated in the *Polymorphisms and Bu Pharmacokinetics* prospective observational multicenter study (NCT01257854). Covariate distributions and doses received by the patients are detailed in **Supplemental Digital Content 1** (see **Supplementary Material S2**, http://links.lww.com/TDM/A747). Bu monitoring in these patients involved extensive sampling at 0, 0.25, 0.5, 1, and 4 hours after infusion end for dose 1, and dose 5, dose 9, or both for q6h dosing. For q24h dosing, concentration measurements were available at 0, 0.5, 1, 2, 3, and 5 hours postinfusion. Patients without drug level measurements on at least 2 different days were excluded from the analysis. In addition, we assessed model performance without *GSTA1* genetic information or IOV. A comparison was added with MAP estimates from a pediatric PopPK model validated for Bu's MIPD by Shukla et al.^21^ The model code from the latter publication, written in Phoenix NLME according to reported parameters,^21^ was utilized for generating MAP estimated cAUC_0–∞_. We also compared MAP model-based estimations of AUC with noncompartmental analysis (NCA)–based estimations obtained using Phoenix WinNonLin with log-linear interpolation for the elimination phase. For q6h patients, only the first dose AUC_0–∞_ was considered in the comparison between MAP and NCA due to the absence of data and the nonfeasibility of monitoring all administered doses in that setting. For q24h patients, as all concentrations for all the administered doses were available, it was possible to compare both the first dose and cumulative AUC_0–∞_ of MAP versus NCA. Furthermore, we estimated the relative MPE and RMSE at the concentrations level (MPE_C_ and RMSE_C_) between the concentrations measured in the patients (reference values) and the MAP-predicted concentrations using the determined optimal sampling scenarios.

### Implementation of the Model in Tucuxi

Tucuxi (Yverdon-les-Bains, Switzerland) is a graphical user interface used for implementing PopPK models for routine model-informed TDM in clinical settings.^26^ A new drug file for Bu was created using Tucuxi's online drug file editor (available from: http://drugeditor.tucuxi.ch/). As the current version of Tucuxi does not handle IOV, the random and residual variabilities of the model parameters were re-estimated without IOV before the creation of the drug file (see **Supplementary Material S3, Supplemental Digital Content 1**, http://links.lww.com/TDM/A747). Following implementation in Tucuxi, we assessed the reliability of the software cAUC_0–∞_ estimations with limited sampling scenarios, by comparing them with the MAP estimations provided by Phoenix NLME software, using Bland–Altman analyses.

## RESULTS

### Simulation-Based Evaluation of Sampling Schedules (Times)

**FIGURE 2.**
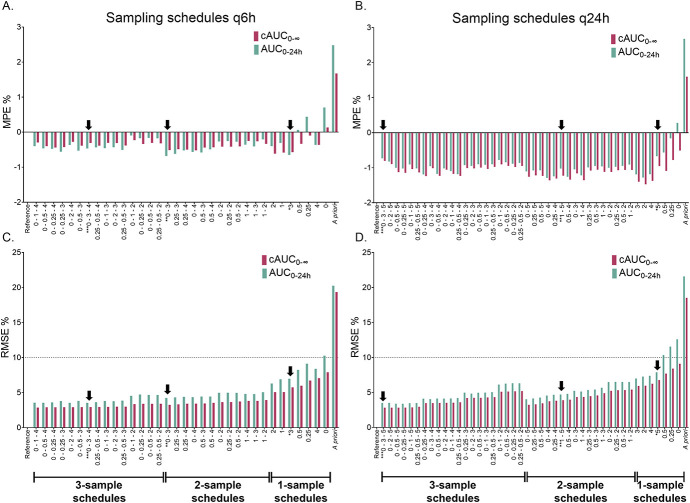
Simulation-based evaluation of limited sampling time points for accuracy (relative mean prediction error: MPE%) and precision (relative root mean square error: RMSE) of MAP estimation of AUC_0–24h_ and cAUC_0–∞_. Schedules are ranked by ascending RMSE % of cAUC_0–∞_. A and C illustrate the results of q6h (4 times daily) dosing, and B and D illustrate the results of q24h (once daily) dosing. Reference values for MPE and RMSE calculations were the full-simulated AUC values. For a priori estimation, no sampling times were input, and only the covariate model was used for calculation. The optimal 3-sample, 2-sample, and 1-sample schedules determined by the PFIM software are indicated by black arrows.

For q6h, PFIM calculation indicated that the optimal 3-sample schedule was at 0–3–4 hours after the infusion end, with 0–3 hours as the optimal 2-sample schedule, and 3 hours as a single-sample schedule. In the case of q24h, the predicted optimal schedules were at 0–3–5 hours after the infusion end for a 3-sample schedule, 1–5 hours for a 2-sample schedule, and 5 hours for a 1-sample schedule. According to the PFIM predictions, the 3-hour time point appeared crucial for parameter estimations in q6h dosing, whereas the 5-hour time point consistently emerged as a critical time point for monitoring q24h dosing. The simulation-based assessment of the sampling schedules is illustrated in Figure 2. All the tested limited sampling schedules exhibited a relative bias of <5%, and many of these schedules demonstrated a RMSE lower than the predefined threshold of 10%. Thus, MIPD with MAP estimation enables flexible sampling times while maintaining acceptable accuracy and precision.

The sampling times predicted by PFIM were among the most accurate and precise schedules, demonstrating the utility of this software in the optimal design of TDM protocols. Several schedules with only 1 sample meet the defined RMSE criteria. The a priori predictions were minimally biased. The precision was also acceptable, considering that the a priori estimations were based solely on the covariate model, without observation input. However, these a priori predictions did not meet the criterion of 10% RMSE, which was expected given the random Bu CL variability. Thus, Bu dosing using a priori estimated parameters may not replace TDM, particularly when targeting the narrow therapeutic window proposed by Bartelink et al.^6^

For the 2-sample and 3-sample schedules, the measurement at time 0 hours (t_max_), right at the end of the infusion, demonstrated slightly improved precision compared with schedules where the first collected sample was delayed. Thus, the peak concentration is important for characterizing the distribution of Bu. In addition, in q24h administration, a final sample taken 5 hours after infusion completion (8 hours after infusion start) consistently improved the estimation precision compared with schedules not including this time point, suggesting that this sampling time is highly informative for estimating AUC. PFIM calculations also supported this result. For 1-sample schedules, the sampling times proposed by PFIM (ie, 3 hours for q6h and 5 hours for q24h) had RMSE <10%, although they were not the most precise. The most precise 1-sample time points were 2 and 3 hours after infusion end for q6h and q24h, respectively.

### Evaluation of Sampling Scenarios (Times and Days)

**FIGURE 3.**
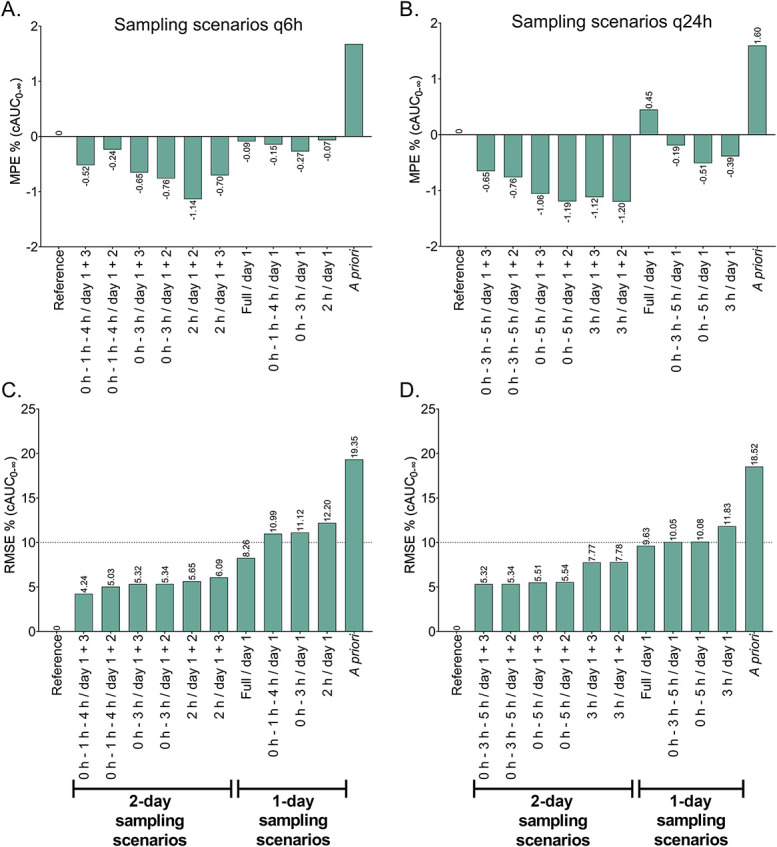
Simulation-based selection of optimal sampling scenarios (limited sampling schedule + day of monitoring) based on accuracy (relative mean prediction error: MPE %) and precision (relative root mean squared error: RMSE) of MAP estimation of cAUC_0–∞_. Scenarios are ranked by ascending RMSE % of cAUC_0–∞_. Reference: full-simulated AUC values. A and C: MPE and RMSE of tested scenarios for q6h dosing. B and D: MPE and RMSE of tested scenarios for q24h dosing.

Following the selection of optimal 1-sample, 2-sample, and 3-sample schedules for both q6h and q24h, various sampling scenarios were assessed for the accuracy and precision of cAUC_0–∞_ estimation, in comparison with simulated values (results shown in Fig. 3). While all tested scenarios fell within an acceptable bias range, monitoring across 2 different days exhibited lower RMSE compared with single-day monitoring, even with extensive sampling on that single day. Most limited sampling scenarios on the first day resulted in RMSE values >10%. Sampling on the first and third days yielded marginally better precision than sampling on the first and second days, possibly due to stabilization of Bu PK parameters from the third day onward. These findings collectively indicate that TDM of Bu with LSS on a single dosing occasion may not suffice, and sampling across at least 2 different days is advisable. Nonetheless, the scenario involving extensive sampling on the first day resulted in borderline acceptable RMSE.

### Validation of the Limited Sampling Scenarios With Real Patients' Data

**FIGURE 4.**
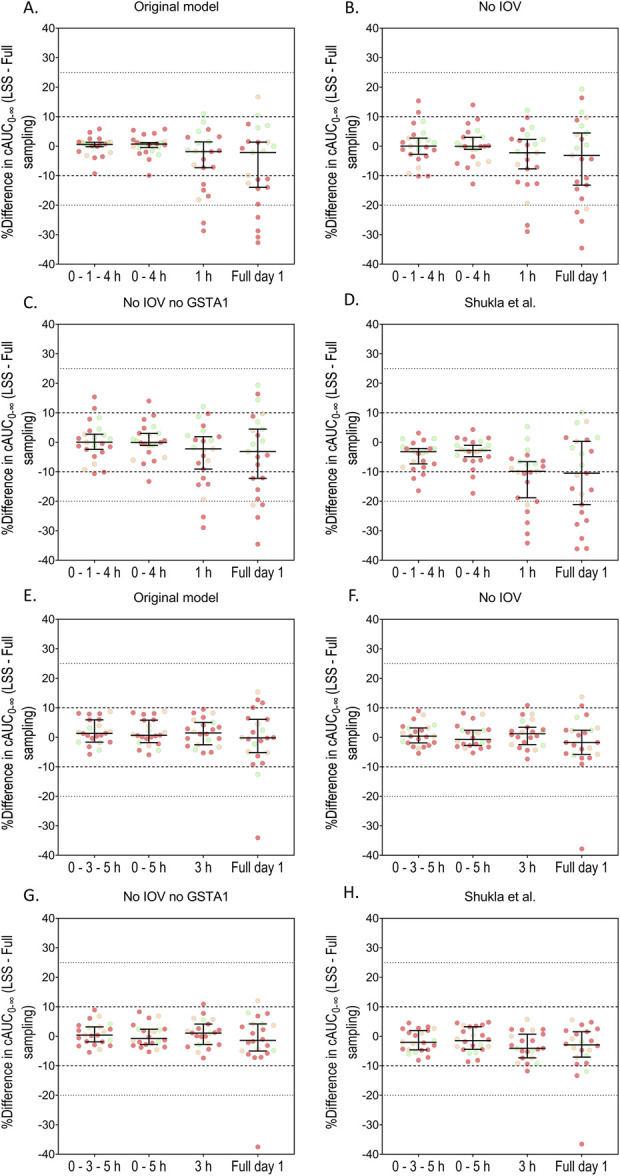
Evaluation of optimal limited sampling scenarios with data from the validation patient cohort. All selected patients were monitored through extensive sampling on at least 2 occasions. Limited sampling schedules included selected time points on either day 1 + day 2 or day 1 + day 3, depending on data availability. Full day 1: full sampling on the first day of Bu administration. Dashed lines represent ±10% deviation from reference value. Dotted lines represent 80%–125% deviation interval. Error bars represent median with 95% CI. Red dots indicate patients with Bu dose modifications >25%; yellow, 10%–25%; green, and less than 10%. Panels A to D display evaluation of q6h scenarios, and panels E to H display evaluation of q24h scenarios.

The validation process evaluated the most precise 1-sample, 2-sample, and 3-sample schedules for q6h and q24h, considering sampling on the first, second, or third day of conditioning, depending on data availability. Because Tucuxi software does not handle IOV, the random effects and residual variability were re-estimated using our previously published model building data set^14^ while removing the IOV. The fixed-effects parameters were not re-estimated. **Supplemental Digital Content 1** (see **Supplementary Material S3**, http://links.lww.com/TDM/A747) summarizes the new parameter estimates. The results obtained using real patient data are shown in Figure 4. Two of the critical sampling times previously discovered at q6h, 2 and 3 hours after infusion end, were not routinely collected in these patients. Thus, we selected the next best performing schedule in terms of RMSE for validation in q6h patients, which was 0–4 hours for the 2-sample schedule and 1 hour for the 1-sample schedule (Fig. 4 A–D). While the one-sample schedule was proven sufficiently precise for the once-daily dosing (q24h), it resulted in considerable variability in q6h patients, with 2 patients deviating from the reference AUC value by more than 20% (Fig. 4 A–C). This may be because the single-sample schedule was not the most optimal schedule determined in our simulations for the q6h scenarios. A sampling time of 2 hours after infusion end is expected to result in improved precision. The model without IOV had acceptable precision, indicating that it could be used in the Tucuxi software (Fig. 4 B and F). The absence of *GSTA1* covariate information did not affect the MAP PK estimations (Fig. 4 C and G). The bias and precision results of the MAP concentrations listed in Table 1 indicate that the proposed LSS strategies are suitable for individual concentration predictions. Higher RMSEc values were nonetheless consistently observed for estimations using models without IOV.

**TABLE 1.** Accuracy and Precision of MAP Systemic Concentration Predictions (MPEc % and RMSEc %, Respectively) With Limited Sampling Scenarios in the Validation Cohort

q6h	0–1–4 h (NObs = 138)		0–4 h (NObs = 92)		1 h (NObs = 46)	
	MPE_C_ %	RMSE_C_ %	MPE_C_ %	RMSE_C_ %	MPE_C_ %	RMSE_C_ %
Original model	−0.5	6.7	−0.9	5.9	−1.6	5.6
No IOV	−0.5	10.3	−0.7	11.7	−2.3	11.2
No IOV no GSTA1	−0.5	10.3	−0.7	11.8	−2.3	11.3
Shukla et al	−2.7	8.9	−3.5	9.6	−4.5	9.8

q24h	0–3–5 h (NObs = 126)		0–5 h (NObs = 84)		3 h (NObs = 42)	
	MPE_C_ %	RMSE_C_ %	MPE_C_ %	RMSE_C_ %	MPE_C_ %	RMSE_C_ %
Original model	−0.5	4.4	−0.6	3.8	−0.6	3.4
No IOV	−0.9	6.4	−1.0	6.7	−1.1	5.6
No IOV no GSTA1	−1.0	6.4	−1.1	6.6	−1.3	5.6
Shukla et al	−2.3	4.7	−2.5	4.9	−3.2	4.5

Reference values for calculations were measured plasma concentrations.

GSTA1, glutathione-S-transferase A1 metabolizing capacity; IOV, random inter-occasion variability; MPEc, relative mean prediction error at concentration level; NObs, number of observations used in calculation; q6h, 4times-daily dosing; q24h, once-daily dosing; RMSEc, relative root mean square error at concentration level.

Expectedly, for both q6h and q24h, a full sampling schedule limited to the first day of conditioning yielded poorer results than those observed with limited sampling on 2 separate days. In particular, for q6h Bu, a scenario based solely on the first day, even with extensive sampling, was insufficiently accurate and precise, regardless of dose modifications. Even patients with dose modifications <10% had inaccurate cAUC_0–∞_ estimations with the first-day-only sampling scenario (Fig. 4 A–D).

Calculations using the model of Shukla et al^21^ yielded similar exposure estimations to those of our model, even when limited schedules were applied (Fig. 4 D and H). The comparison between NCA-based and model-based estimation of AUC is presented in **Supplemental Digital Content 1** (see **Supplementary Materials S4 and S5**, http://links.lww.com/TDM/A747) and showed a good concordance between MAP and NCA first dose and cumulative AUC_0–∞_ estimations. For q6h dosing, significant extrapolated portions in NCA might be the cause of the differences observed between MAP and NCA first dose AUC_0–∞_. MAP tended to slightly overestimate the AUC compared with NCA in q24h dosing and underestimated it in q6h (see **Supplementary Materials S4 and S5, Supplemental Digital Content 1**, http://links.lww.com/TDM/A747).

### Implementation of the Model in *Tucuxi* Software

The model without IOV and with the re-estimated interindividual variability and residual error was implemented in the Tucuxi software. A comparison of Tucuxi and Phoenix estimates is shown in **Supplemental Digital Content 1** (see **Supplementary Materials S6**, http://links.lww.com/TDM/A747). Excellent concordance was observed between the values estimated by Tucuxi and Phoenix, demonstrating that the use of Tucuxi in the routine MIPD of Bu is reliable. An example of patient interpretation using Tucuxi is presented in **Supplemental Digital Content 1** (see **Supplementary Materials S7**, http://links.lww.com/TDM/A747).

## DISCUSSION

We developed a protocol for TDM of Bu in pediatric patients employing the Bayesian MAP approach along with a LSS. Furthermore, we explored the optimal TDM scenarios, considering the days of monitoring, through model-based simulations. The validation of these findings was conducted using real patient data for the 2 prevalent administration schedules of Bu: q24h or q6h.

Research has delved into the LSS of Bu (see **Supplementary Materials S8, Supplemental Digital Content 1**, http://links.lww.com/TDM/A747). In this study, alongside the model-assisted LSS proposition, we underscore the necessity of TDM on at least 2 occasions to precisely target the cumulative AUCs of Bu. The simplest LSS methodology relies on multiple linear regression models correlating the measured concentrations at specific times with the AUC. However, this approach hinges on the accuracy of the measured concentrations and can be sensitive to variations in sampling times and/or measurement errors, rendering their routine implementation challenging.^23,27,28^ Furthermore, linear regression models may be overfitted to the model development data set and display a lack of external applicability. The use of Bayesian MAP estimation with PopPK models for TDM offers distinct advantages in mitigating inaccuracies associated with sampling times and concentration measurements. MAP estimation provides a robust method for determining the most probable individual PK parameter values by combining prior knowledge (eg, population estimates) with observed data.^29^ This integrated approach helps to compensate for potential deviations in sampling times and concentration measurements, leading to more reliable and accurate predictions of drug exposure in individual patients,^30–32^ as demonstrated for Bu by Dadkhah et al.^29^ This population-based approach enables estimation of individual PK parameters of drugs, even with limited, irregular, or inaccurately reported sampling times.^29,33^ However, the Bayesian approach with LSS is not reliable when the patient displays outlying concentrations and substantially deviates from the distribution of the population model parameters.^23^ Comprehensive studies on the Bu LSS that systematically evaluate sampling scenarios in terms of sampling times and days for both q6h and q24h are lacking.

A challenge in designing this study was determining the most suitable MPE and RMSE thresholds for selecting LSS schedules. Guidelines for these thresholds are lacking. El Hassani et al suggested that these thresholds should be tailored to the specific characteristics of the drug under investigation, particularly its therapeutic window.^34^ We opted for thresholds of ±5% for MPE and 10% for RMSE to ensure accurate achievement of the narrow therapeutic window proposed by Bartelink et al.^6^ This window is centered at 90 mg.h/L, with a range of 78–101 mg.h/L, representing an approximate ±10% deviation from the central value.^6^

One challenge we faced was selecting the most appropriate reference AUC value to validate the LSS scenarios. For instance, while estimating cAUC_0–∞_ by NCA requires full sampling at every administered dose, this extensive sampling is only feasible for q24h dosing. In addition, NCA is more sensitive to errors in concentration measurements, potentially leading to flawed estimations of AUC_0–∞_, particularly when a high percentage of extrapolation to infinity is involved.^32^ Generally, AUC_0–∞_ estimation is deemed unreliable when extrapolation exceeds 20%–25% of the total AUC.^35^ In our study, we encountered this issue, particularly with q6h dosing, where the final measurement could only be taken 6 hours after the start of infusion. Nevertheless, we compared AUC estimations derived from NCA and MAP methods (see **Supplementary Materials S4 and S5, Supplemental Digital Content 1**, http://links.lww.com/TDM/A747). Still, for validation purposes, we prioritized comparing AUC_0–∞_ obtained with full sampling against LSS scenarios. Furthermore, we evaluated the MPE and RMSE of the model-predicted versus measured concentrations, revealing accurate and precise individual concentration predictions using the LSS (Table 1).

For q6h dosing, a minimum of 2 samples is required per monitored dose, ideally taken at 0 and 3 hours after the infusion ends. Conversely, a single sample, preferably taken 3 hours after the infusion ends, is adequate for accurately estimating MAP with q24h dosing. Reliable cumulative AUC estimation necessitates TDM on 2 separate days for both dosing regimens, preferably on the first and third days of Bu administration. The selection of optimal time points is crucial for capturing individual PK characteristics and maximizing the accuracy and precision of MAP PK parameter estimation. With q6h dosing, characterized by more frequent Bu administration, multiple peaks and troughs in drug concentrations occur over the 4-day treatment period. This frequent dosing and resultant concentration fluctuations present challenges in selecting appropriate time points for concentration measurement. In addition, accumulation of Bu was observed at q6h dosing, but not at q24h. Bu concentrations reach steady state around 12 hours after the first administration (Bu elimination half-life ≈2.5 hours^36^), that is, from the third q6h dose onward. As previously noted, a single concentration measurement proved insufficient for adequately capturing individual Bu PK parameters in this instance. Conversely, employing 2 time points for concentration measurement with q6h dosing yielded a better appraisal of the steady-state concentrations and more accurately captured the fluctuating drug concentrations. By contrast, once-daily dosing exhibits less fluctuation in drug concentrations compared with q6h dosing. Therefore, a single time point for concentration measurement proved adequate for reliably estimating once-daily dosing, as the drug concentrations remained relatively stable. To our knowledge, this study marks the first evaluation of the LSS for 2 different dosing schedules of Bu commonly employed in clinical settings.

Our results also suggest that the TDM of Bu solely on the first day of conditioning is insufficient to accurately estimate the cumulative exposure, especially in q6h dosing, even when rich sampling is employed on the first measured dose. The same observations were made in our analyses using the Shukla et al model,^21^ indicating that this result is not specific to our model. Either day 1 and 2 or day 1 and 3 sampling scenarios could be employed, as both of these scenarios result in accurate and precise cAUC_0–∞_ estimations. In both scenarios, dose adjustment based on the results obtained on day 1 (estimated AUC_0–24h_) would mitigate potential punctual over/underexposure. A subsequent TDM course conducted on either day 2 or 3 would enable fine-tuning of the dose to achieve the desired cumulative exposure. For transplantation units lacking immediate access to TDM laboratories (eg, where dose recommendations cannot be obtained on the same day), sampling on the first 2 days of conditioning would be advisable to provide sufficient time for required dose adjustments to be made.

A study by Bognàr et al^18^ reported that Bu monitoring on both days 1 and 2 does not yield a significant difference from monitoring on day 1 alone in terms of variance in CL on the fourth day. However, monitoring on both days could potentially reduce the variance in exposure. Our results indicate that due to random unexplained intraindividual variability in CL, even a model considering a decrease in CL on subsequent conditioning days cannot substitute for additional monitoring of Bu on different days. Consequently, we advocate for a second TDM of Bu to be performed either on day 2 or 3 of conditioning, irrespective of dose modification. Despite the typical decrease in CL of 5.8%–12% in pediatric patients,^14–17,20^ patients may experience a greater extent of CL decrease or even a CL increase for a minority of them, as previously reported.^15,37^ Although the exact mechanism underlying this variability remains unclear, variations in the reservoir of glutathione available for Bu conjugation have emerged as a likely hypothesis.^19^ A recent study showed that EdAG, a glutathione analog generated during Bu metabolism, depletes cysteine and glutathione in vitro,^38^ potentially contributing to the observed decrease in Bu CL. Moreover, the hypothesis of autoinhibition of Bu metabolism cannot be excluded.^39^

Overall, factors clearly explaining this random variability between occasions are still lacking, and a second course of TDM on day 2 or 3 of treatment should be performed to guarantee the achievement of the therapeutic target. Future research must explore the factors explaining this random intraindividual variability and identify reliable biomarkers, such as endogenous proteomic or metabolomic markers, that correlate with Bu elimination. The integration of these markers into MIPD is required to further reduce the need for Bu TDM. This type of work was recently performed by McCune et al,^40^ focusing on the predose metabolomic profile to personalize the initial dose of Bu. Interestingly, several endogenous metabolomic compounds discovered are involved in the glutathione synthesis pathway.^40^ We propose that exploring the relevance of these markers for predicting Bu CL variations and integrating them as covariates in PK prediction models may be a further step toward limiting the need for multiple-day sampling in Bu TDM.

Although this study suggests that MIPD of Bu with LSS may be beneficial for optimizing dosing in pediatric patients, it is important to consider the feasibility and cost-effectiveness of this approach in clinical practice. Future studies should explore the practical considerations involved in implementing the MIPD of Bu in clinical settings and compare the costs and benefits of LSS with those of extensive sampling strategies. The benefits of LSS should also be clearly assessed.

## CONCLUSIONS

We established optimal MIPD protocols for administering Bu once- and 4-times-daily in pediatric patients. These protocols entail collecting a very limited number of samples over 2 separate days. Relying solely on TDM conducted after the first dose of Bu is insufficient for accurately estimating the cumulative AUC of Bu, even with extensive sampling of the initial dose. While establishing a protocol for MIPD of Bu has been our focus, it is crucial to consider the potential impact of this approach on clinical outcomes in pediatric patients. Prospective evaluation of the proposed precision dosing setup for Bu, which includes individualized initial dosing considering *GSTA1* polymorphisms along with TDM using our optimized sampling, is necessary to validate this workflow and further enhance the efficiency of achieving target exposure levels of Bu. Future studies should investigate whether the MIPD strategy for Bu improves clinical outcomes, such as reducing toxicity and increasing survival rates.

## Supplementary Material

SUPPLEMENTARY MATERIAL

## References

[1] Klyuchnikov E Langebrake C Badbaran A Individualized busulfan dosing improves outcomes compared to fixed-dose administration in pre-transplant minimal residual disease-positive acute myeloid leukemia patients with intermediate-risk undergoing allogeneic stem cell transplantation in CR. Eur J Haematol. 2023;110:188–197. 36335432 10.1111/ejh.13893

[2] Gurlek Gokcebay D Arman Bilir O Şahin S . Role of therapeutic drug monitoring of intravenous Busulfan for prevention of sinusoidal obstructive syndrome in children. Pediatr Transplant. 2022;26:e14266. 35343635 10.1111/petr.14266

[3] Hill BT Rybicki LA Urban TA Therapeutic dose monitoring of busulfan is associated with reduced risk of relapse in non-Hodgkin Lymphoma patients undergoing autologous stem cell transplantation. Biol Blood Marrow Transplant. 2020;26:262–271. 31610237 10.1016/j.bbmt.2019.09.033

[4] Faraci M Tinelli C Lanino E Monitoring of busulphan concentrations in children undergone hematopoietic stem cell transplantation: unicentric experience over 10 years. Eur J Drug Metab Pharmacokinet. 2018;43:173–181. 28801891 10.1007/s13318-017-0431-0

[5] Salman B Al-Za’abi M Al-Huneini M Therapeutic drug monitoring-guided dosing of busulfan differs from weight-based dosing in hematopoietic stem cell transplant patients. Hematol Oncol Stem Cell Ther. 2017;10:70–78. 28408108 10.1016/j.hemonc.2017.03.003

[6] Bartelink IH Lalmohamed A van Reij EML Association of busulfan exposure with survival and toxicity after haemopoietic cell transplantation in children and young adults: a multicentre, retrospective cohort analysis. Lancet Haematol. 2016;3:e526–e536. 27746112 10.1016/S2352-3026(16)30114-4 PMC5159247

[7] Esteves I Santos FPS Ribeiro AAF Targeted-dose of busulfan: higher risk of sinusoidal obstructive syndrome observed with systemic exposure dose above 5000 µMol.min. A historically controlled clinical trial. Hematol Oncol. 2020;38:773–781. 32779746 10.1002/hon.2789

[8] Andersson BS Thall PF Valdez BC Fludarabine with pharmacokinetically guided IV busulfan is superior to fixed-dose delivery in pretransplant conditioning of AML/MDS patients. Bone Marrow Transplant. 2017;52:580–587. 27991894 10.1038/bmt.2016.322 PMC5382042

[9] Chen T Chen C He X . Fixed-dose administration and pharmacokinetically guided adjustment of busulfan dose for patients undergoing hematopoietic stem cell transplantation: a meta-analysis and cost-effectiveness analysis. Ann Hematol. 2022;101:667–679. 35091794 10.1007/s00277-021-04733-3

[10] Combarel D Tran J Delahousse J . Individualizing busulfan dose in specific populations and evaluating the risk of pharmacokinetic drug-drug interactions. Expert Opin Drug Metab Toxicol. 2023;19:75–90. 36939456 10.1080/17425255.2023.2192924

[11] Briki M André P Thoma Y Precision oncology by point-of-care therapeutic drug monitoring and dosage adjustment of conventional cytotoxic chemotherapies: a perspective. Pharmaceutics. 2023;15:1283. 37111768 10.3390/pharmaceutics15041283 PMC10147065

[12] Lawson R Staatz CE Fraser CJ . Review of the pharmacokinetics and pharmacodynamics of intravenous busulfan in paediatric patients. Clin Pharmacokinet. 2021;60:17–51. 33128207 10.1007/s40262-020-00947-2

[13] Ben Hassine K Powys M Svec P Total body irradiation forever? Optimising chemotherapeutic options for irradiation-free conditioning for paediatric acute lymphoblastic leukaemia. Front Pediatr. 2021;9:775485. 34956984 10.3389/fped.2021.775485 PMC8705537

[14] Ben Hassine K Nava T Théoret Y Precision dosing of intravenous busulfan in pediatric hematopoietic stem cell transplantation: results from a multicenter population pharmacokinetic study. CPT Pharmacometrics Syst Pharmacol. 2021;10:1043–1056. 34453497 10.1002/psp4.12683 PMC8452291

[15] Marsit H Philippe M Neely M Intra-individual pharmacokinetic variability of intravenous busulfan in hematopoietic stem cell-transplanted children. Clin Pharmacokinet. 2020;59:1049–1061. 32157629 10.1007/s40262-020-00877-z

[16] Bartelink I Boelens JJ Bredius RGM Body weight-dependent pharmacokinetics of busulfan in paediatric haematopoietic stem cell transplantation patients: towards individualized dosing. Clin Pharmacokinet. 2012;51:331–345. 22455797 10.2165/11598180-000000000-00000

[17] McCune JS Bemer MJ Barrett JS . Busulfan in infant to adult hematopoietic cell transplant recipients: a population pharmacokinetic model for initial and Bayesian dose personalization. Clin Cancer Res. 2014;20:754–763. 24218510 10.1158/1078-0432.CCR-13-1960 PMC3946385

[18] Bognàr TT Kingma JSJ Smeijsters EHE Busulfan target exposure attainment in children undergoing allogeneic hematopoietic cell transplantation: a single day versus a multiday therapeutic drug monitoring regimen. Bone Marrow Transplant. 2023;58:762–768. 37002411 10.1038/s41409-023-01971-z

[19] Langenhorst JB Boss J van Kesteren C A semi-mechanistic model based on glutathione depletion to describe intra-individual reduction in busulfan clearance. Br J Clin Pharmacol. 2020;86:1499–1509. 32067250 10.1111/bcp.14256 PMC7373715

[20] Lawson R Staatz CE Fraser CJ Population pharmacokinetic model for once-daily intravenous busulfan in pediatric subjects describing time-associated clearance. CPT Pharmacometrics Syst Pharmacol. 2022;11:1002–1017. 35611997 10.1002/psp4.12809 PMC9381908

[21] Shukla P Goswami S Keizer RJ Assessment of a model-informed precision dosing platform use in routine clinical care for personalized busulfan therapy in the pediatric hematopoietic cell transplantation (HCT) population. Front Pharmacol. 2020;11:888. 32714184 10.3389/fphar.2020.00888 PMC7351521

[22] Dupuis LL Sibbald C Schechter T IV busulfan dose individualization in children undergoing hematopoietic stem cell transplant: limited sampling strategies. Biol Blood Marrow Transplant. 2008;14:576–582. 18410900 10.1016/j.bbmt.2008.03.002

[23] de Jonge ME Huitema ADR Schellens JHM . Individualised cancer chemotherapy: strategies and performance of prospective studies on therapeutic drug monitoring with dose adaptation: a review. Clin Pharmacokinet. 2005;44:147–173. 15656695 10.2165/00003088-200544020-00002

[24] Paci A Vassal G Moshous D Pharmacokinetic behavior and appraisal of intravenous busulfan dosing in infants and older children: the results of a population pharmacokinetic study from a large pediatric cohort undergoing hematopoietic stem-cell transplantation. Ther Drug Monit. 2012;34:198–208. 22406655 10.1097/FTD.0b013e31824c2f60

[25] Dumont C Lestini G Le Nagard H PFIM 4.0, an extended R program for design evaluation and optimization in nonlinear mixed-effect models. Comput Methods Programs Biomed. 2018;156:217–229. 29428073 10.1016/j.cmpb.2018.01.008

[26] Dubovitskaya A Buclin T Schumacher M . TUCUXI: an intelligent system for personalized medicine from individualization of treatments to research databases and back. In: Proceedings of the 8th ACM International Conference on Bioinformatics, Computational Biology, and Health Informatics. ACM-BCB '17. New York, NY, USA: Association for Computing Machinery; 2017:223–232.

[27] Ting LSL Villeneuve E Ensom MHH. Beyond cyclosporine: a systematic review of limited sampling strategies for other immunosuppressants. Ther Drug Monit. 2006;28:419–430. 16778729 10.1097/01.ftd.0000211810.19935.44

[28] Sarem S Nekka F Ahmed IS . Impact of sampling time deviations on the prediction of the area under the curve using regression limited sampling strategies. Biopharm Drug Dispos. 2015;36:417–428. 25845479 10.1002/bdd.1951

[29] Dadkhah A Alihodzic D Broeker A . Evaluation of the robustness of therapeutic drug monitoring coupled with Bayesian forecasting of busulfan with regard to inaccurate documentation. Pharm Res. 2021;38:1721–1729. 34664209 10.1007/s11095-021-03115-8 PMC8602150

[30] Stifft F Vandermeer F Neef C . A limited sampling strategy to estimate exposure of once-daily modified release tacrolimus in renal transplant recipients using linear regression analysis and comparison with Bayesian population pharmacokinetics in different cohorts. Eur J Clin Pharmacol. 2020;76:685–693. 32020321 10.1007/s00228-019-02814-x

[31] Marquet P Bedu A Monchaud C Pharmacokinetic therapeutic drug monitoring of Advagraf in more than 500 adult renal transplant patients, using an expert system online. Ther Drug Monit. 2018;40:285–291. 29505493 10.1097/FTD.0000000000000503

[32] Alsultan A An G Peloquin CA. Limited sampling strategy and target attainment analysis for levofloxacin in patients with tuberculosis. Antimicrob Agents Chemother. 2015;59:3800–3807. 25870068 10.1128/AAC.00341-15 PMC4468713

[33] Cho SH Lee JH Lim HS Prospective validation of a novel dosing scheme for intravenous busulfan in adult patients undergoing hematopoietic stem cell transplantation. Korean J Physiol Pharmacol. 2016;20:245–251. 27162478 10.4196/kjpp.2016.20.3.245 PMC4860366

[34] El Hassani M Marsot A. External evaluation of population pharmacokinetic models for precision dosing: current state and knowledge gaps. Clin Pharmacokinet. 2023;62:533–540. 37004650 10.1007/s40262-023-01233-7

[35] Gabrielsson J Weiner D. Non-compartmental analysis. In: Reisfeld B Mayeno AN, eds *Computational Toxicology*. Vol I. Methods in Molecular Biology. Totowa, NJ: Humana Press; 2012:377–389. 10.1007/978-1-62703-050-2_16 23007438

[36] European Medicines Agency. Busulfan Fresenius-Kabi Product Information; 2021. Available at: https://www.ema.europa.eu/en/documents/product-information/busulfan-fresenius-kabi-epar-product-information_en.pdf. Accessed September 20, 2023.

[37] Alsultan A Albassam AA Alturki A Population pharmacokinetics of busulfan in Saudi pediatric patients undergoing hematopoietic stem cell transplantation. Int J Clin Pharm. 2020;42:703–712. 32140913 10.1007/s11096-020-00989-3

[38] Hoang S Dao N Myers AL. Electrophilic reactivity of the Busulfan metabolite, EdAG, towards cellular thiols and inhibition of human thioredoxin-1. Biochem Biophys Res Commun. 2020;533:325–331. 32958252 10.1016/j.bbrc.2020.09.038

[39] Hassan M Ljungman P Ringdén O The effect of busulphan on the pharmacokinetics of cyclophosphamide and its 4-hydroxy metabolite: time interval influence on therapeutic efficacy and therapy-related toxicity. Bone Marrow Transplant. 2000;25:915–924. 10800057 10.1038/sj.bmt.1702377

[40] McCune JS Navarro SL Baker KS Prediction of busulfan clearance by predose plasma metabolomic profiling. Clin Pharmacol Ther. 2023;113:370–379. 36369996 10.1002/cpt.2794 PMC9888309

