# Supplementary material for: Simulation-Based Optimization of Sampling Schedules for Model-Informed Precision Dosing of Once-Daily and 4-Times-Daily Busulfan in Pediatric Patients
Source: Ther Drug Monit. 2024 Jun 14;46(6):786–96. doi: 10.1097/FTD.0000000000001217 (PMC11554249; doi:10.1097/FTD.0000000000001217)
Supplement: SUPPLEMENTARY MATERIAL [file tdm-46-786-s001.docx]

Supplementary material

S1. Demographics and summary of the doses administered for the development (simulation) cohort.

S2. Demographics and summary of the doses administered for the validation cohort (NCT01257854)

S3. Update of PopPK model random and residual variability parameters in absence of IOV

S4. Comparison between NCA and MAP estimated first dose AUC_0-∞_ for q24h and q6h dosing

S5. Comparison between NCA and MAP estimated cumulative AUC_0-∞_ for q24h dosing only.

S6. Comparison of cAUC0-∞ obtained with Phoenix software with cAUC0-∞ obtained with Tucuxi software, using real patient data.

S7. Example of patient TDM interpretation with Tucuxi software

S8. Literature review of published limited sampling strategies for Bu.

**S1. Demographics (A) and summary of the doses administered (B) for the development (simulation) cohort.**

A)

| **Patient characteristics** | | **N (%)** | **Median (Range)** |
| --- | --- | --- | --- |
| **Total** |  | **100** |  |
| **Sex** | Male | 56 (56) |  |
|  | Female | 44 (44) |  |
| **Age (years)** |  |  | 6.0 (0.2–20.0) |
| **Weight (kg)** |  |  | 19.1 (4.5–86.0) |
|  | 0–10 kg | 18 (18) |  |
|  | 10–20 kg | 35 (35) |  |
|  | 20–30 kg | 10 (10) |  |
|  | 30–50 kg | 20 (20) |  |
|  | > 50 kg | 17 (17) |  |
| **BMI (kg/m^2^)** |  |  | 16.6 (10.4–29.6) |
| **Bu dosing schedule** | Q6H | 35 (35) |  |
|  | Q24H | 65 (65) |  |
| **Administered doses (mg/kg)** | Q6H |  | 0.97 (0.51 – 1.61) |
|  | Q24H |  | 3.79 (0.96 – 8.00) |
| **Cond. regimen** | Non-Flu regimens | 56 (56) |  |
|  | Flu regimens | 44 (44) |  |
| ***GSTA1* group** | 1 | 18 (18) |  |
|  | 2 | 64 (64) |  |
|  | 3 | 18 (18) |  |

Cond.: Conditioning; Flu: fludarabine; Q6H: four-times daily Bu dosing; Q24H: once-daily Bu dosing;

B)

Each line represents a single patient.

II: Interval between the doses in hours.

**S2. Demographics (A) and summary of the doses administered (B) for the validation cohort (NCT01257854)**

A)

| **Patient characteristics** | | **N (%)** | **Median (Range)** |
| --- | --- | --- | --- |
| **Total** |  |  |  |
| **Sex** | Male | 23 (51) |  |
|  | Female | 22 (49) |  |
| **Age (years)** |  |  | 7.8 (0.3–18.3) |
| **Weight (kg)** |  |  | 21.6 (5.5–68.2) |
|  | 0–10 kg | 6 (13) |  |
|  | 10–20 kg | 14 (31) |  |
|  | 20–30 kg | 4 (9) |  |
|  | 30–50 kg | 14 (31) |  |
|  | > 50 kg | 7 (16) |  |
| **BMI (kg/m^2^)** |  |  | 16.9 (11.9–23.9) |
| **Bu dosing schedule** | Q6H | 23 (51) |  |
|  | Q24H | 22 (49) |  |
| **Administered doses (mg/kg)** | Q6H |  | 0.79 (0.45 – 1.61) |
|  | Q24H |  | 4.03 (2.07 – 8.16) |
| **Cond. regimen** | **Non-Flu regimens** | 29 (64) |  |
|  | **Flu regimens** | 16 (36) |  |
| ***GSTA1* group** | 1 | 6 (13) |  |
|  | 2 | 29 (64) |  |
|  | 3 | 10 (22) |  |

Cond.: Conditioning; Flu: fludarabine; Q6H: four-times daily Bu dosing; Q24H: once-daily Bu dosing;

B)

Each line represents a single patient.

II: Interval between the doses in hours.

**S3. Update of PopPK model random variability (expressed as CV%) and residual error parameters in absence of IOV**

|  | Previous parameters | Updated parameters without IOV |
| --- | --- | --- |
| IIV CL (%) | 17 | 22 |
| IIV V_1_ (%) | 11 | 17 |
| IIV Q (%) | 66 | 64 |
| IIV V_2_ (%) | 64 | 70 |
| IOV CL (%) | 13 | **NA** |
| IOV V (%) | 14 | **NA** |
| Proportional residual error (%) | 6 | 11 |

**S4. Comparison between NCA and MAP estimated first dose AUC_0-∞_ for q24h and q6h dosing**

1. q6h dosing

1. q24h dosing

**S5. Comparison between NCA and MAP estimated cumulative AUC_0-∞_ for q24h dosing only.**

**S6. Comparison of cAUC_0-∞_ obtained with Phoenix software with cAUC_0-∞_ obtained with Tucuxi software, using real patient data.**


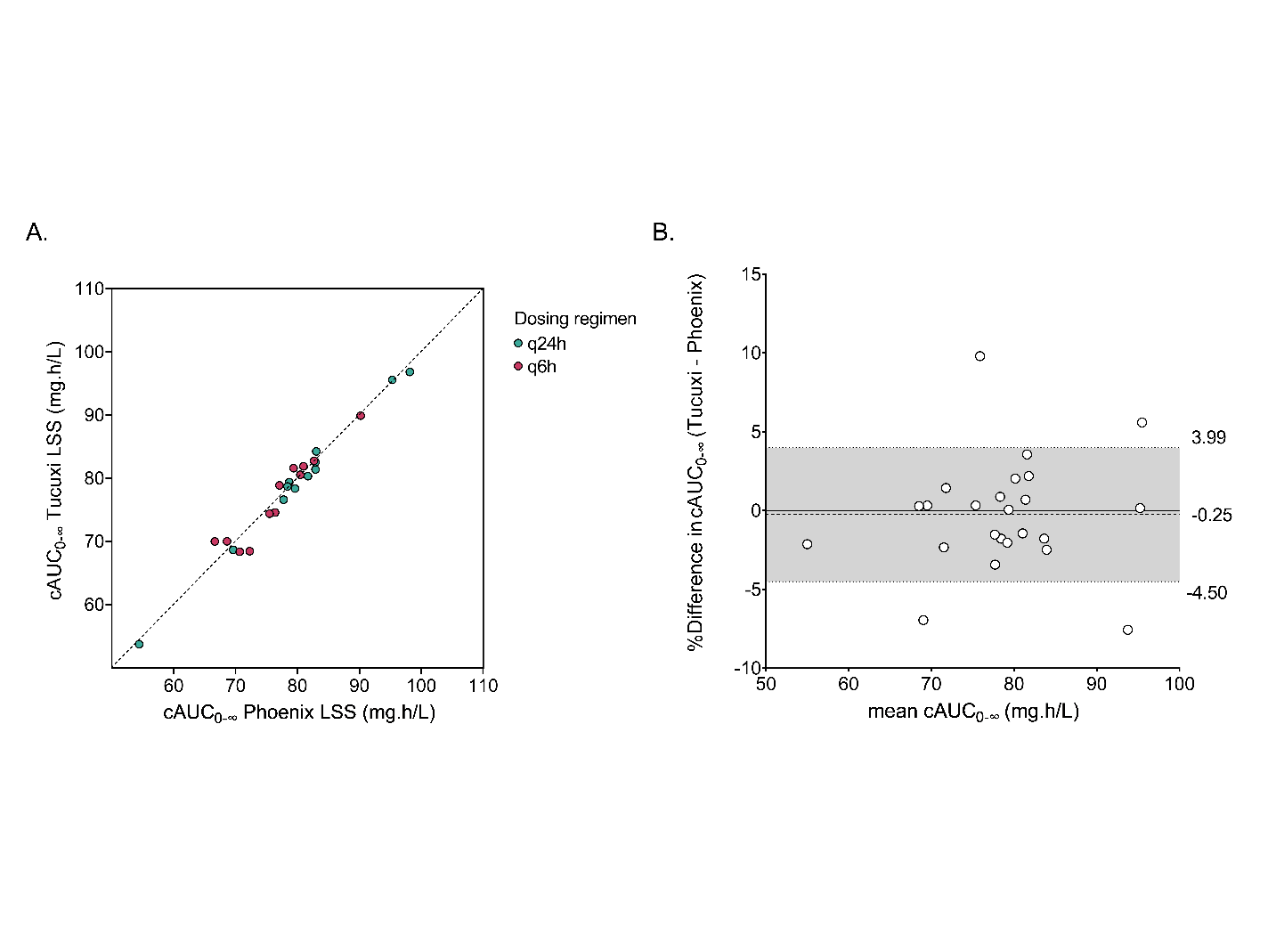


A: Tucuxi vs. Phoenix generated cAUC_0-∞_. Dashed line indicates identity line. B: Bland–Altman plot for difference in cAUC_0-∞_ estimation between Phoenix and Tucuxi software. Solid line represents identity line, dashed line represents mean bias, and shaded area represents 95% limit of agreement.

**
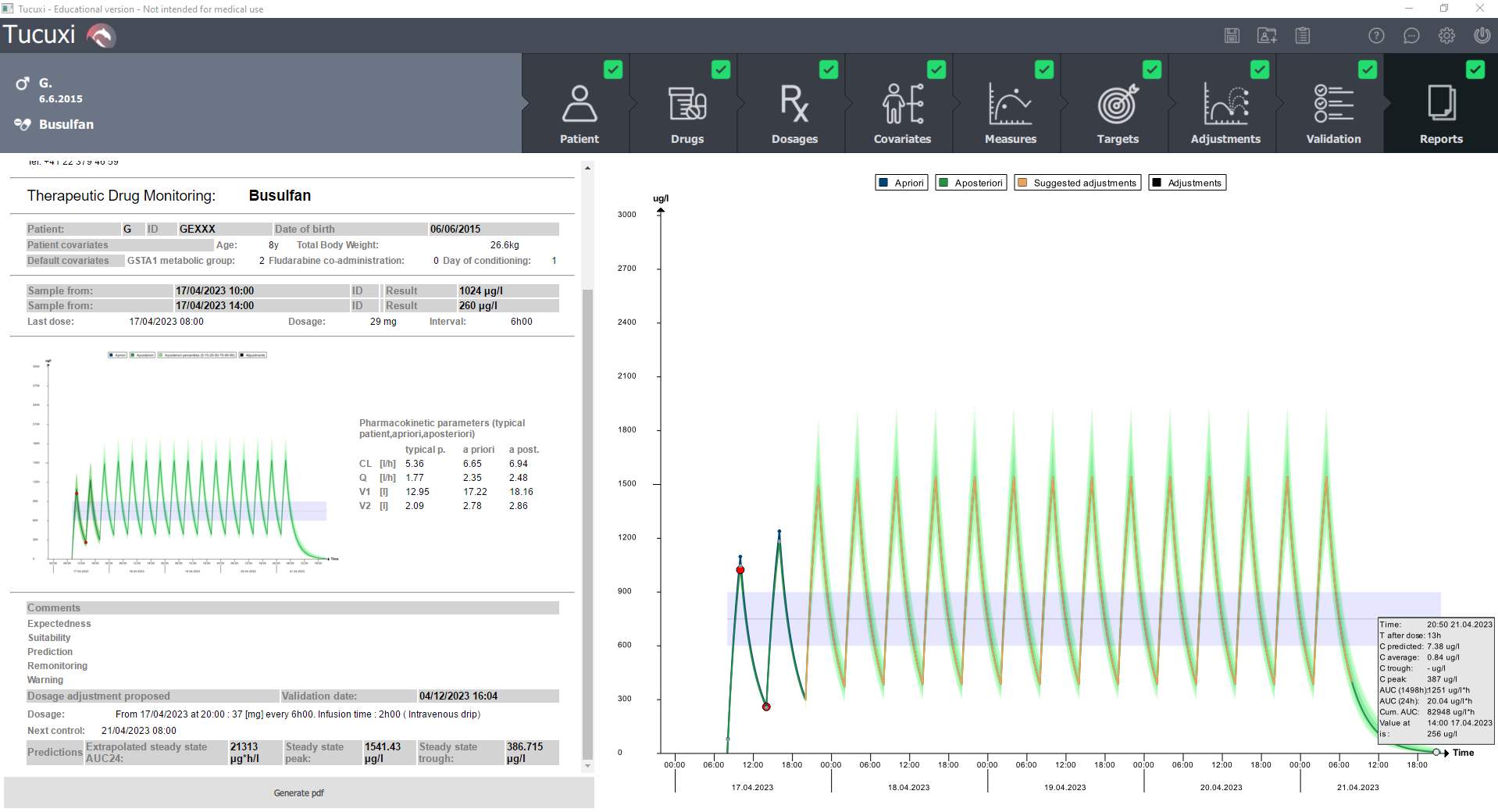
S7. Example of patient TDM interpretation with Tucuxi software**

**S8. Literature review of published limited sampling strategies evaluated for IV busulfan.**

| **Study reference** | **Population**  **(N, Age range or median ± SD)** | **Dosing schedule** | **Analytical method(s) (with LLOQ if available)** | **LSS model** | **Reference parameter for LSS validation** | **N of samples** | **Sampling schemes** | **MPE** | **RMSE** | **Other criterium if RMSE not reported** | **Requirement of a specific Software** |
| --- | --- | --- | --- | --- | --- | --- | --- | --- | --- | --- | --- |
| Cremers et al. ^1^ | model development:  N = 6  Age: 1.5 – 14 years  LSS validation:  1000 simulated subjects with fixed dosing  0.8 mg/kg/6h | Four times daily | HPLC-UV  After precolumn derivatization and LLE  LLOQ: 30 ng/ml | Bayesian maximum a posteriori estimation with PopPK model | First dose AUC calculated from Simulated Dose and CL  (AUC = Dose/CL) | 2 | 2.5 and 6 h | NR | NR | Pearson correlation (predicted vs. observed)  R = 0.970 | PopPK modelling software. |
| Dupuis et al.^2^ | Model development:  N = 44  Age: 0.3 – 16.2  Model validation:  N = 35  Age: 0.4 – 18.3 years | Four times daily | GC-ECD  LLOQ: NR | Linear regression models | First dose AUC calculated by NCA | 3 | 4, 5 and 6 h | -0.03% | 2.3% | NA | None required |
|  |  |  |  |  |  | 2 | 4 and 5 h | -0.05% | 2.5% |  |  |
|  |  |  |  |  |  | 1 | 5 h | 0.17 % | 3.9% |  |  |
| Huang et al. ^3^ | N = 45  Age: 31 ± 11 years  No separate validation cohort | Twice daily | HPLC-MS/MS  LLOQ :  0.02 µmol/L = 5 ng/ml | Linear regression models | AUCs of Dose 1 and Dose 7 calculated by NCA | 3 | 2, 3 and 6 h | 0.44% | NR | MAPE: 5.47% | None required |
|  |  |  |  |  |  | 2 | 3 and 6 h | 0.52% | NR | MAPE:  6.32% |  |
|  |  |  |  |  |  | 1 | 3 h | 0.88% | NR | MAPE:  8.37 % |  |
| Goutelle et al. ^4^ | N = 178  Age: 0.13 – 21 years | Four times daily | HPLC-UV or HPLC with fluorescence detector and pre-column derivatization.  Linearity range: 20 – 2000 ng/ml^5^. | Bayesian maximum a posteriori estimation with PopPK model from Paci et al.^6^ (without IOV) | Observed (measured) concentrations | 2 | 2.5 h and 4h | 6.4% | 11.4% | NA | Model implemented in Tucuxi Software (freely available) |
| Kishimoto et al. ^7^ | N = 5  Age: 2 – 12 years  The LSS was tested on Additional 1000 simulated patients of 1, 3, 10 and 30 kg | Four times daily | HPLC-MS/MS  LLOQ: NR | Bayesian maximum a posteriori estimation based on McCune et al.^8^ model | Cumulative AUC obtained from Bayesian forecasting  Based on a full set of samples (5 samples) on measured form the first administered dose | 3 | 2, 6 and 24 h | -8.6 % | NR | MAPE: 8.6% | PopPK modelling software. McCune et al. model is also available in a Web-based TDM software from Nextdose.org |
|  |  |  |  |  |  | 2 | 6 and 24 h | 0.8 % | NR | MAPE: 5.8% |  |
|  |  |  |  |  |  | 1 | 6 h | 1.3 % | NR | MAPE: 6.1% |  |
| Lawson et al.^9^ | N = 32  Age: 0.2 – 16.5 years | Once daily | UPLC-MS/MS  After July 2014  LLOQ = 10 ng/ml  Before July 2014  HPLC-UV  LLOQ: 100 ng/ml | Bayesian maximum a posteriori estimation using InsightRX software (Shukla et al.^10^ model)  Or NextDose software (McCune et al.^8^ model) | Cumulative AUC  AUC_0-24h_  AUC_0-inf_ of the fourth dose, computed by NCA | 3 | Pre-dose, 3 and 8 h | (Median relative error)  InsightRX:  AUCcum:  10.1 %  AUC_0-24_:  9.8%  NextDose:  AUC_cum_:  3.4%  AUC0-24:  4.5% | InsightRX:  AUCcum:  0.20 mg.h/L  AUC_0-24_:  0.18 mg.h/L  NextDose:  AUC_cum_:  0.10 mg.h/L  AUC0-24:  0.27 mg.h/L | NA | Avialable in Web-based TDM software.  Shukla et al. ^10^ and McCune et al. ^8^ models can also be written in a modelling software to obtain Post-hoc parameters |
| Neely et al. ^5^ | Model development:  N=53  Age: 0.2 – 19.0 years  BestDose software validation cohort.  N=20  Age: 0.25 – 18 years | Four times daily | HPLC-UV or HPLC with fluorescence detector and pre-column derivatization.  Linearity range: 20 – 2000 ng/ml. | Estimation using Multiple Model method based on a Non-parametric population model | Doses predicted to achieve a target C_ave_ | 2 | 2.25 h and 6 h | Dose 3:  1.9%  Dose 7:  -0.7%  Dose 11:  4.0% | Bias corrected RMSE :  Dose 3 :  4.6%  Dose 7:  9.9%  Dose 11:  12.1% | NA | BestDose software. The model can also be written in Pmetrics package for R. |
|  |  |  |  |  |  | 1 | 2.25 h | Dose 3:  -9.2 %  Dose 7:  -10.8%  Dose 11:  -11.1% | Dose 3 :  15.7%  Dose 7:  16.4%  Dose 11:  22.1% | NA |  |
| Nguyen et al. ^11^ | Model development:  N=103  Age: 19.0 – 64.0 years  Model validation:  N=24  Age: 14.3 – 64.0 years | Four times daily | GC-MS  LLOQ: NR  LOD: 62.5 ng/ml | Bayesian maximum a posteriori estimation forecasting with PopPK model | AUC_0-inf_ (dose 1) and AUC_0-t_ (dose 9) | 2 | 2.25h and 6 h | -0.32% | 5.9% | NA | PopPK modelling software. |
|  |  |  |  |  |  | 1 | 3 h | -0.94% | 6.9% |  |  |
| Utano et al. ^12^ | Model development:  N=54  Age: 0.2 – 19.2 years  Model validation:  N=7  Age: 0.9 – 8.3 years | Four times daily | HPLC-MS/MS  LLOQ: 10 ng/mL | Linear regression models | AUC_0-inf_ of the first dose determined by NCA | 3 | 3h, 4h and 6h | NR | NR | MAPE: 5.9%  R^2^: 0.955 | None required |
|  |  |  |  |  |  | 2 | 3h and 6h | NR | NR | MAPE: 6.4%  R^2^: 0.943 |  |
|  |  |  |  |  |  | 1 | 6h | NR | NR | MAPE: 11.0%  R^2^: 0.789 |  |
| Watanabe et al.^13^ | Model development:  N=29  Age: 0.7 – 17.0 years  No separate validation cohort | Four times daily | HPLC-UV  LLOQ: NR | Linear regression models | AUC_0-inf_ of the first dose determined by NCA | 3 | 2h, 3h and 6h | -1.04% | 7.1% | NA | None required |
|  |  |  |  |  |  | 2 | 2h and 6h | -0.46% | 7.5% |  |  |
|  |  |  |  |  |  | 1 | 6h | 0.28 | 7.9% |  |  |
| Yuan et al.^14^ | Model development:  N=69  Age: 0.5 – 15.2 years  Model validation:  N= 14  Age: 0.6 – 12.6 years | Four times daily | HPLC-MS/MS  LLOQ: 10 ng/mL | Bayesian maximum a posteriori estimation with PopPK model | AUC_0-6h_ obtained with Bayesian forecasting  Based on a full set of samples (5 samples) form the first dose | 3 | 2h, 2.25h, and 4h | NR | 0.7% | NA | PopPK modelling software. |
|  |  |  |  |  |  | 2 | 2h and 4h | NR | 1.0% |  |  |

AUC: Area under the concentration curve; C_ave_: average concentration; CL: Clearance; ECD: electron capture detection; GC: Gas chromatography; HPLC: High performance liquid chromatography; LOD: Lower limit of detection; LLOQ: Lower limit of quantification; MAPE: Mean absolute prediction error; MPE: Mean prediction error; MS: Mass spectrometry detection; MS/MS: tandem mass spectrometry detection; NA: Not applicable; NCA: Noncompartmental analysis; NR: Not reported; PopPK: Population pharmacokinetics; RMSE: Root-mean-square error, UPLC: Ultra-performance liquid chromatography; UV: Ultraviolet detection.

**References:**

1. Cremers S, Schoemaker R, Bredius R, et al. Pharmacokinetics of intravenous busulfan in children prior to stem cell transplantation. *Br J Clin Pharmacol*. 2002;53(4):386-389. doi:10.1046/j.1365-2125.2002.01555.x

2. Dupuis LL, Sibbald C, Schechter T, et al. IV busulfan dose individualization in children undergoing hematopoietic stem cell transplant: limited sampling strategies. *Biol Blood Marrow Transplant J Am Soc Blood Marrow Transplant*. 2008;14(5):576-582. doi:10.1016/j.bbmt.2008.03.002

3. Huang J jing, Chen B, Hu J, Yang WH. Limited sampling strategy for predicting busulfan exposure in hematopoietic stem cell transplantation recipients. *Int J Clin Pharm*. 2017;39(4):662-668. doi:10.1007/s11096-017-0481-z

4. Goutelle S, Thoma Y, Buffet R, et al. Implementation and Cross-Validation of a Pharmacokinetic Model for Precision Dosing of Busulfan in Hematopoietic Stem Cell Transplanted Children. *Pharmaceutics*. 2022;14(10):2107. doi:10.3390/pharmaceutics14102107

5. Neely M, Philippe M, Rushing T, et al. Accurately Achieving Target Busulfan Exposure in Children and Adolescents With Very Limited Sampling and the BestDose Software. *Ther Drug Monit*. 2016;38(3):332-342. doi:10.1097/FTD.0000000000000276

6. Paci A, Vassal G, Moshous D, et al. Pharmacokinetic Behavior and Appraisal of Intravenous Busulfan Dosing in Infants and Older Children: The Results of a Population Pharmacokinetic Study From a Large Pediatric Cohort Undergoing Hematopoietic Stem-Cell Transplantation. *Ther Drug Monit*. 2012;34(2):11.

7. Kishimoto K, Hasegawa D, Irie K, et al. Pharmacokinetic analysis for model-supported therapeutic drug monitoring of busulfan in Japanese pediatric hematopoietic stem cell transplantation recipients. *Pediatr Transplant*. 2020;24(4):e13696. doi:10.1111/petr.13696

8. McCune JS, Bemer MJ, Barrett JS, Scott Baker K, Gamis AS, Holford NHG. Busulfan in infant to adult hematopoietic cell transplant recipients: a population pharmacokinetic model for initial and Bayesian dose personalization. *Clin Cancer Res Off J Am Assoc Cancer Res*. 2014;20(3):754-763. doi:10.1158/1078-0432.CCR-13-1960

9. Lawson R, Paterson L, Fraser CJ, Hennig S. Evaluation of two software using Bayesian methods for monitoring exposure and dosing once-daily intravenous busulfan in paediatric patients receiving haematopoietic stem cell transplantation. *Cancer Chemother Pharmacol*. May 2021. doi:10.1007/s00280-021-04288-0

10. Shukla P, Goswami S, Keizer RJ, et al. Assessment of a Model-Informed Precision Dosing Platform Use in Routine Clinical Care for Personalized Busulfan Therapy in the Pediatric Hematopoietic Cell Transplantation (HCT) Population. *Front Pharmacol*. 2020;11. doi:10.3389/fphar.2020.00888

11. Nguyen L, Leger F, Lennon S, Puozzo C. Intravenous busulfan in adults prior to haematopoietic stem cell transplantation: a population pharmacokinetic study. *Cancer Chemother Pharmacol*. 2006;57(2):191-198. doi:10.1007/s00280-005-0029-0

12. Utano T, Kato M, Sakamoto K, et al. Two-point blood sampling is sufficient and necessary to estimate the area under the concentration-time curve for intravenous busulfan in infants and young children. *Pediatr Blood Cancer*. April 2021:e29069. doi:10.1002/pbc.29069

13. Watanabe E, Nishikawa T, Ikawa K, et al. Trough level monitoring of intravenous busulfan to estimate the area under the plasma drug concentration-time curve in pediatric hematopoietic stem cell transplant recipients. *Int J Hematol*. 2015;102(5):611-616. doi:10.1007/s12185-015-1853-6

14. Yuan J, Sun N, Feng X, et al. Optimization of Busulfan Dosing Regimen in Pediatric Patients Using a Population Pharmacokinetic Model Incorporating GST Mutations. *Pharmacogenomics Pers Med*. 2021;14:253-268. doi:10.2147/PGPM.S289834
